# DOCK2: A novel FLT3/ITD leukemia drug target

**DOI:** 10.18632/oncotarget.21390

**Published:** 2017-09-30

**Authors:** Min Wu, Donald Small, Amy S. Duffield

**Affiliations:** Amy S. Duffield: Department of Pathology, Sidney Kimmel Comprehensive Cancer Center, The Johns Hopkins Hospital, Baltimore, MD, USA

**Keywords:** acute myeloid leukemia, FMS-like tyrosine kinase-3 (FLT3), internal tandem duplication, DOCK2, Rac1

Acute myeloid leukemia (AML) is an aggressive hematologic malignancy characterized by the clonal expansion of immature myeloid cells in the bone marrow and other tissues. Approximately one third of AML cases carry a mutation in the FMS-like tyrosine kinase-3 (FLT3) receptor gene. FLT3 plays important roles in the survival and proliferation of hematopoietic stem/progenitor cells. The most frequent FLT3 mutation is an internal tandem duplication (ITD) in the juxtamembrane domain, which leads to constitutive autophosphorylation and activation of downstream signaling pathways, including PI-3-kinase/AKT, RAS/ERK and STAT5. AML patients who carry a FLT3/ITD mutation have a relatively poor prognosis [1].

Since tyrosine kinase inhibitors have shown great promise in the treatment of other hematopoietic neoplasms such as chronic myeloid leukemia, FLT3 is an appealing drug target. To that end, there has been a steady influx of potential FLT3 inhibitors. While these inhibitors are able to induce clinical remissions in many patients, most ultimately relapse and long-term inhibition of FLT3 activity remains a challenge [2]. Since a more complete understanding of normal and mutant FLT3 biology may help develop a strategy to more effectively target this pathway, we performed a screen to identify a panel of proteins that interact with FLT3. Numerous candidate interactors were identified, one of which was dedicator of cytokinesis 2 (DOCK2) [3].

DOCK2 is a member of the DOCK family of proteins. The DOCK proteins act as guanine nucleotide exchange factors (GEFs) for Rho GTPases, which regulate critical cellular functions and have been shown to play important roles in cancer progression. DOCK2 specifically activates both Rac1 and Rac2. Rac1 is known to regulate several crucial processes, including lymphocyte migration, activation and differentiation of T cells, cell-cell adhesion and bone marrow homing of some immune cells. The potential importance of Rac1 in FLT3/ITD AML has been suggested in work by Sallmyr et al, who showed that Rac1 is activated by FLT3/ITD and facilitates the generation of reactive oxygen species and DNA damage in leukemic cells [4].

Rac1 has been considered as a potential therapeutic target in a variety of neoplasms, but since this protein is so widely expressed and affects disparate cellular functions — including glucose uptake by skeletal muscle — the side effect profile is likely to be unacceptable. Thus, DOCK2 provides an interesting and novel drug target for reducing Rac1 activity in leukemic cells, since the expression of this GEF is limited to hematolymphoid tissues, which should limit the off-target effects.

Our study confirmed that DOCK2 is expressed in hematopoietic cell lines and primary AML patient samples, and that it interacts with both wild-type FLT3 and FLT3/ITD in these cells [3]. Knock-down (KD) of DOCK2 was accomplished via shRNA. DOCK2 KD in FLT3/ITD cells resulted in a concomitant decrease in both the activity of STAT5 and the generation of reactive oxygen species. Additionally, decreased DOCK2 expression resulted in markedly reduced cell proliferation and colony formation in cell lines with elevated FLT3 activity. DOCK2 KD also rendered these cells significantly more sensitive to cytarabine (Ara-C), a chemotherapeutic agent that is widely used in both induction and consolidation regimens in AML treatment. In contrast, cell lines lacking FLT3 activity displayed no observable difference when DOCK2 expression was suppressed. Interestingly, the Ara-C sensitivity of a FLT3/ITD cell line (MV4;11) with DOCK2 KD was further enhanced in the presence FLT3 inhibitors. Furthermore, mice transplanted with MV4;11 cells showed markedly prolonged leukemia survival when DOCK2 was knocked down, with a median survival of 55.5 days, as compared to 15 days in mice transplanted with control MV4;11 cells.

These findings suggest that DOCK2 and the Rac1 pathway may play a critical role in cell proliferation in FLT3/ITD AML. To date, the role of DOCK2 in both hematological malignancies and normal hematopoiesis has not been extensively studied. Much of the research has focused on the role of DOCK2 in the immune system, since DOCK2 mutations have been found in patients with immunodeficiencies [5]. Other studies have focused on B-cell homing and migration as well as B-cell lymphomas. For example, Wang, et al, found that DOCK2 played a role in the regulation of cell proliferation in a B-cell lymphoma via the ERK signaling pathway [6].

Our findings implicate DOCK2 as a potential drugable target for AML with FLT3/ITD mutations. Additionally, they show that although RhoGTPases may not be feasible drug targets due to their widespread expression and protean functions, inhibition of more tissue specific upstream GEFs may allow for the development of drugs that inhibit the Rho GTPases with an acceptable side effect profile. Although DOCK2 inhibitors are not commercially available, several small molecular inhibitors of DOCK2 have been reported [7]. More efforts in the development of DOCK2 inhibitors may be warranted, as DOCK2 inhibition — when coupled with FLT3 inhibitors and conventional chemotherapeutic agents — may provide a new target for the treatment of FLT3/ITD AML.

## References

[1] Small D (2006). Hematology Am Soc Hematol Educ Program.

[2] Small D (2008). Semin Hematol.

[3] Wu M (2017). Leukemia.

[4] Sallmyr A (2008). Blood.

[5] Dobbs K (2015). N Engl J Med.

[6] Wang L (2010). Biochem Biophys Res Commun.

[7] Nishikimi A (2013). Exp Cell Res.

